# Detection of skewed X-chromosome inactivation in Fragile X syndrome and X chromosome aneuploidy using quantitative melt analysis

**DOI:** 10.1017/erm.2015.11

**Published:** 2015-07-01

**Authors:** David E. Godler, Yoshimi Inaba, Charles E. Schwartz, Quang M. Bui, Elva Z. Shi, Xin Li, Amy S. Herlihy, Cindy Skinner, Randi J. Hagerman, David Francis, David J. Amor, Sylvia A. Metcalfe, John L. Hopper, Howard R. Slater

**Affiliations:** 1 Cyto-molecular Diagnostic Research Laboratory, Victorian Clinical Genetics Services and Murdoch Children's Research Institute, Royal Children's Hospital, Melbourne, Victoria, 3052, Australia; 2 Center for Molecular Studies, J.C. Self Research Institute of Human Genetics, Greenwood Genetic Center, South CA, USA; 3 Centre for Molecular, Environmental, Genetic and Analytic Epidemiology, University of Melbourne, Carlton, Victoria, 3053, Australia; 4 Public Health Genetics, Murdoch Children's Research Institute, Royal Children's Hospital, Melbourne, Victoria, 3052, Australia; 5 Andrology Australia, Clayton, Victoria, 3168, Australia; 6 The MIND Institute, University of California, Davis Medical Center, Sacramento, CA, USA; 7 Department of Pediatrics, University of California, Davis School of Medicine, Sacramento, CA, USA; 8 Department of Paediatrics, University of Melbourne, Melbourne Victoria, 3052, Australia; 9 Genetics Education and Health Research, Murdoch Children's Research Institute, Royal Children's Hospital, Melbourne, Victoria, 3052, Australia

## Abstract

Methylation of the fragile X mental retardation 1 (*FMR1*) exon 1/intron 1 boundary positioned fragile X related epigenetic element 2 (FREE2), reveals skewed X-chromosome inactivation (XCI) in fragile X syndrome full mutation (FM: CGG > 200) females. XCI skewing has been also linked to abnormal X-linked gene expression with the broader clinical impact for sex chromosome aneuploidies (SCAs). In this study, 10 FREE2 CpG sites were targeted using methylation specific quantitative melt analysis (MS-QMA), including 3 sites that could not be analysed with previously used EpiTYPER system. The method was applied for detection of skewed XCI in FM females and in different types of SCA. We tested venous blood and saliva DNA collected from 107 controls (CGG < 40), and 148 FM and 90 SCA individuals. MS-QMA identified: (i) most SCAs if combined with a Y chromosome test; (ii) locus-specific XCI skewing towards the hypomethylated state in FM females; and (iii) skewed XCI towards the hypermethylated state in SCA with 3 or more X chromosomes, and in 5% of the 47,XXY individuals. MS-QMA output also showed significant correlation with the EpiTYPER reference method in FM males and females (*P* < 0.0001) and SCAs (*P* < 0.05). In conclusion, we demonstrate use of MS-QMA to quantify skewed XCI in two applications with diagnostic utility.

## Introduction

Abnormal DNA methylation is an important cause of aberrant regulation of gene expression of relevance in a large number of pathologies. For high-throughput, targeted, locus specific, quantitative methylation analysis, the Matrix-assisted laser desorption/ionisation time of flight mass spectrometry (MALDI-TOF MS) EpiTYPER system has been suggested to have the greater diagnostic potential than other techniques, reviewed in: (Refs 1, 2). MALDI-TOF MS is capable of a technical detection limit as low as 5% and a single nucleotide resolution of the majority of CpG sites within amplicons as large as 0.6 kilobases. Its closest rival is pyrosequencing, which is restricted to much smaller regions and has lower throughput (Ref. 1). Recently, we have used the MALDI-TOF MS based EpiTYPER system to characterise new epigenetic biomarker regions – Fragile X Related Epigenetic Elements (Ref. 3). We have shown the potential clinical utility of these biomarkers in fragile X syndrome (FXS) diagnostics (Refs 4, 5, 6), a major inherited condition co-morbid with autistic behaviours and intellectual disability, reviewed in: (Ref. 7). We have also validated EpiTYPER analyses of these biomarkers on DNA derived from venous blood, saliva, adult and newborn dried blood spots (Ref. 8), and have shown fragile X mental retardation 1 (*FMR1*) full mutation (FM) expansions in females (FM: >200 CGG triplet repeats) to be significantly associated with skewed X-inactivation. This however, was not the case for females with *FMR1* premutation expansions (PM: 55–199 repeats) (Ref. 6). We have also demonstrated that these analyses are predictive of cognitive impairment in FXS males and females (Ref. 4), and can be used to detect sex chromosome aneuploidies (SCAs) when combined with a test for sex determining region Y (SRY) copy number (Ref. 8).

However, a major limitation of MALDI-TOF MS is that it requires specialised training and proprietary equipment (Refs 2, 9), which is not available in most molecular diagnostic laboratories. Another limitation is that of the 10 CpG biomarker sites within the fragile X related epigenetic element 2 (FREE2) amplicon, the EpiTYPER approach cannot analyse three sites because they cluster as one fragment, which is too big in size (Dalton) to be captured within the mass spectrum. For this reason, we have developed a low-cost, high-throughput real-time polymerase chain reaction (PCR) and high resolution melt (HRM) based method for quantitative methylation analysis, which we have named methylation specific – quantitative melt analysis (MS-QMA). Since the MS-QMA does not rely on DNA fragmentation, it can be used to analyse methylation of all CpG sites within the FREE2 amplicon.

There are a number of published HRM based methods developed for quantitative methylation analysis for locus specific (Refs 10, 11, 12, 13) or genome wide (Ref. 14) applications, each with advantages and limitations for their specific applications. However, one common feature between all of these is use of methylation standards generated from unmethylated samples spiked at different ratios with artificially methylated DNA to derive in different ways methylation percentage for the unknown samples. An important distinction between these methods and MS-QMA is that MS-QMA uses a real-time PCR based internal filter process on serially diluted bisulphite converted DNA to ‘clean’ the data prior to a methylation value being derived. Although, we have found that this filter process is essential for the applications described in this study, this was not performed in previously described quantitative HRM methods, which were primarily developed for assessments in the low methylation range in cancer diagnostics (Refs 10, 11, 12, 13). The lack of a filtering/quality control process post-conversion could also be also the reason quantitative HRM has had limited applications for poor quality DNA samples, with none being applied to saliva DNA or archival dried blood spots (Refs 15, 16).

We have used this method to detect FM males and females using venous blood and newborn dried blood spot samples (Ref. 17). However, MS-QMA detection of skewed X-chromosome inactivation (XCI) in different forms of SCA, and in FM females, has not been previously examined. In this study, using a larger cohort, we have compared MS-QMA performance with that of the EpiTYPER system for both saliva and venous blood for these applications as well as for methylation analysis of FM males.

## Materials and methods

### Participants

The patient cohort comprised 245 DNA from venous blood, and 100 saliva DNA samples, with the participants’ age range between birth and 82 years. Of these, 90 samples were from individuals with a SCA or an SRY translocation, 148 were from FM carriers (males and females with a FM allele) and 107 were from controls (with each subgroup and participant age range described in online Supplementary Table 1 for saliva and blood DNA, respectively, with no individuals providing both saliva and blood DNA for this study). EpiTYPER system analysis of all of the samples included in this study has been performed as part of our previous studies (Refs 6, 8). The majority of the samples included in this study were collected as part of FXS cascade testing and routine molecular microarray testing through Victorian Clinical Genetics Services (VCGS) and the Greenwood Genetic Center as described previously (Ref. 8). They included 33 venous blood and four saliva DNA samples from SCAs. All of these samples were de-identified before use in this study. Fifty-three additional SCA individuals who provided saliva DNA were recruited across Australia as part of another study (Ref. 18). An additional 51 FM blood samples collected from carriers were recruited through the VCGS and the M.I.N.D. Institute, University of California at Davis Medical Center, Sacramento, from families seen at the Fragile X Treatment and Research Center through a collaborative Genotype-Phenotype NICHD funded study. The study was approved by the Royal Children's Hospital and Southern Health and Monash University Human Research Ethics Committees, both from Victoria, Australia, and by the Institutional Review Board of the University of California at Davis.

### Molecular studies

#### Sample processing

Venous blood samples of 3–10 ml were collected in Ethylenediaminetetraacetic acid (EDTA)-treated tubes. DNA was extracted using NucleoSpin^®^Tissue genomic DNA extraction kit, as per manufacturer's instructions (MACHEREY-NAGEL GmbH & Co. KG, Düren, Germany); 2 ml saliva samples were collected using the Oragene^®^ DNA Self-Collection Kit (DNA Genotek Inc., Ottawa,Canada) and isolated as per manufacturer's instructions.

#### CGG sizing, methylation using Southern blot and SRY real-time PCR

For venous blood and saliva DNA, the processing of DNA samples and assessment of CGG repeat size (with precision of +/− one repeat) was conducted using a validated PCR amplification assay (Ref. 19). CGG repeat sizing and methylation of the *FMR1* CpG island restriction sites of all samples greater than 55 repeats was also performed using a methylation sensitive Southern blot procedure with appropriate normal and abnormal controls, as previously described (Ref. 20). FREE2 methylation analysis using the Sequenom EpiTYPER system for each sample was performed in quadruplicate, giving four separate methylation output ratios (MOR), which were averaged to take account for technical variation resulting from bisulphite conversion, PCR and mass cleave reactions, as previously described (Refs 3, 5). For SCA samples and control males and females, the SRY copy number was determined using the real-time PCR relative standard curve method, normalised to β-globin, as described in (Ref. 8).

#### MS-QMA

Blood and saliva DNA extracts were treated with sodium bisulphite using EZ-96 DNA Methylation-Gold™ (Zymo Research, Irvine, CA) as previously described (Ref. 8). The 96 well plates of converted DNA were serially diluted four times in water with all dilutions transferred into a 384 well format for MS-QMA analysis of FREE2 biomarkers performed as previously described (Ref. 17). Briefly, real-time PCR portion of MS-QMA utilised MeltDoctor™ high-resolution melt reagents in 10 ul reactions as per manufacturer's instructions (Life technologies, Foster City, CA), using primers targeting specific CpG sites within the FREE2 region, as described in (Ref. 17). The ViiA™ 7 Real-Time PCR System (Life technologies, Foster City, CA) was used for real-time PCR with 65°C the annealing temperature ran for 40 cycles. The dynamic linear range was determined to be between 0.05–10 ng/μl from the standard curve using a series of doubling dilutions of a converted DNA standard from a control lymphoblast cell line. The HRM step followed the real-time PCR for sample within this dynamic linear range in closed tube format. During the HRM step as strands separated the MeltDoctor™ dye was released and detected by the system. The HRM Software Module for ViiA™ 7 System was then used to extract arbitrary fluorescence units (AFU) at 78°C (the temperature that provided the greatest difference in AFU between methylated and unmethylated products). This AFU value was then used to derive methylation ratio for unknown samples from an HRM methylation standard curve co-run on each 384 well plate. This methylation curve was plotted from AFU values over expected methylation of spiked lymphoblast DNA samples from a control male with completely unmethylated FREE2 and a FXS male with 100% methylated FREE2.

#### MS-QMA quality control (QC) and mean methylation ratio (MR)

The AFU values from HRM of the standard curve samples for real-time PCR were used to determine the quality control range. The quality control range represented HRM values where AFU output at 78°C melt did not change between dilutions. The AFU values for the unknown samples with high DNA quality post conversion had to be within this range for their MR values to pass QC filter process. From the four dilutions of each bisulphite conversed DNA sample being assayed, there would be a minimum of 2 and a maximum of 4 MR values that pass QC. The final MR value per sample would then be determined as a mean of all MR from bisulphite converted DNA dilutions for that sample that passed QC.

### Data analysis

The relationship between MALDI-TOF MS CpG10-12 methylation as a predictor and MS-QMA MR as an outcome variable was assessed using simple linear regression analysis. Testing for normality distribution of the methylation ratio for pooled data and each sub-sample was conducted using Shapiro–Wilk test at the significance level of *P* = 0.05. Depending on results of this test for the inter-group comparisons, either two-sample t-test for the means was used, if the data were normally distributed, or nonparametric Mann–Whitney test for median was used, if the data were not normally distributed. All analyses were conducted using the publicly available R statistical computing package (R Development Core Team 2007 R: A Language and Environment for Statistical Computing, R Foundation for Statistical Computing, Vienna, Austria, ISBN 3-900051-07-0. URL: http://www.r-project.org/; date of accession: February 19, 2009).

## Results

### MS-QMA analysis of saliva and venous blood DNA for detection of SCAs

**Figure 1.**
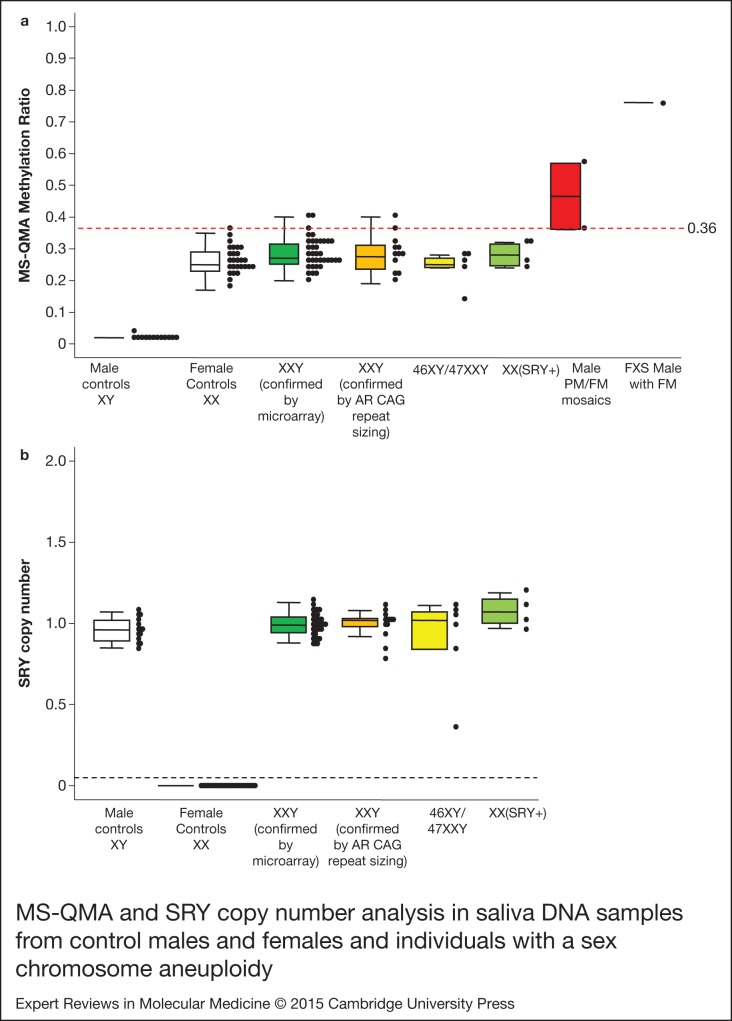
MS-QMA and SRY copy number analysis in saliva DNA samples from control males and females and individuals with a sex chromosome aneuploidy. There were 13 46,XY control males (<40 CGGs); 27 46,XX control females (<40 CGGs); 36 47,XXY Klinefelter syndrome males (confirmed by microarray analysis); 12 47,XXY treated as Klinefelter syndrome males (confirmed by androgen receptor methylation analysis and androgen receptor CAG repeat length heterozygocity; no karyotype available); 5 mosaics for 46,XY/47,XXY (confirmed by microarray analysis); and 4 46,XX males with an SRY translocation (confirmed by microarray analysis), 2 PM/FM size mosaic males, 1 FM male (a) The MR values were determined using MS-QMA; (b) The SRY/β-globin copy number ratios were determined using real-time PCR relative standard curve method. Note: the red broken line represents the maximum value of the female control group; the black broken line represents the optimal threshold value for detection of presence of one or more copies of Y chromosome. FM, full mutation; MR, methylation ratio; MS-QMA, methylation specific quantitative melt analysis; PCR, polymerase chain reaction; PM/FM, permutation/full mutation; SRY, sex determining region Y.

In our earlier study using venous blood samples we have shown that the MS-QMA threshold range between 0.39 and 0.41 MR provided sensitivity and specificity approaching 100% for detection of FM females with verbal IQ (VIQ) impairment (<70) (Ref. 17). The threshold of 0.37 MR was the maximum value of the female healthy control range as well as the optimal for detection of FM females with performance IQ (PIQ) and full scale IQ (FSIQ) impairment (<70). Consistent with this, both blood and saliva DNA from female controls showed MR below the 0.37 maximum control value. Three out of 57 XXY saliva samples (5%) showed MS-QMA MR above the 0.37 MR threshold. Being above the methylation maximum in control females, representing expected range values for ‘normal pattern’ of X-inactivation for 2 X chromosomes in one cell, it suggests that these 5% of XXY samples have extremely skewed X-inactivation in saliva, or presence of cells with a third X chromosome not detected by microarray technology (Fig. 1a). It is also of interest that the saliva DNA of the FXS affected males had MS-QMA MR values well above these thresholds, whereas for the PM/FM mosaic group one male showed an MR of 0.37.

**Figure 2.**
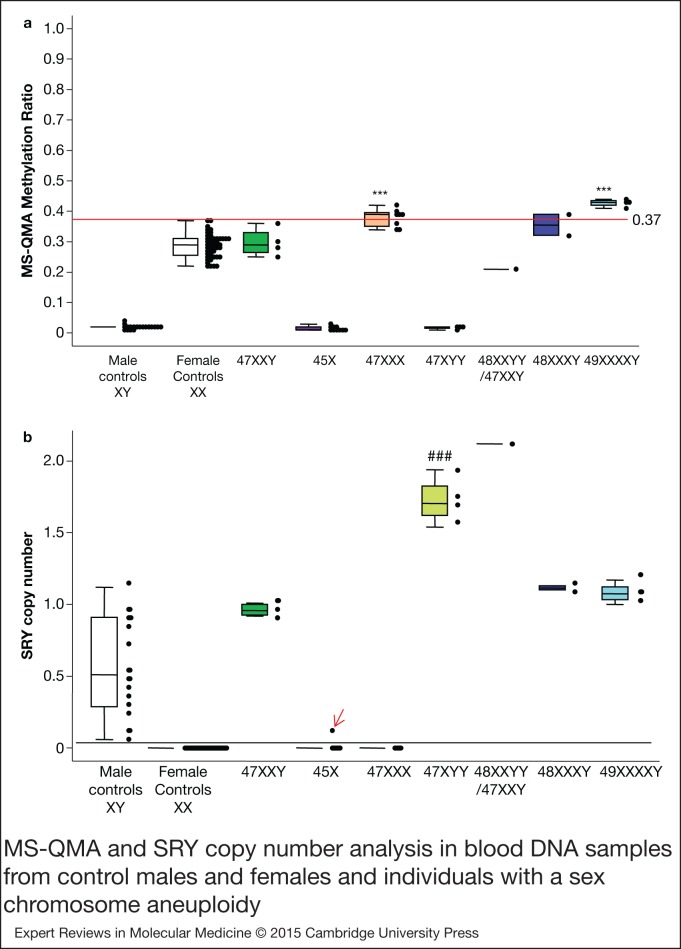
MS-QMA and SRY copy number analysis in blood DNA samples from control males and females and individuals with a sex chromosome aneuploidy. There were 19 46,XY control males (<40 CGGs); 48 46,XX control females (<40 CGGs); 4 47,XXY Klinefelter syndrome males; 10 45,X Turners syndrome females; 8 47,XXX females; 4 47,XYY males; 1 48,XXYY/47,XXY mosaic male; 2 48,XXXY males; and 4 49,XXXXY males. All were confirmed by microarray analysis. (a) The MR values determined using MS-QMA; (b) The *SRY* / β-globin copy number ratios determined using real-time PCR relative standard curve method. Note: the red broken line represent the maximum value of the female control group; the black broken line is the threshold value that optimally separates samples with one or more copies of the Y chromosome. Red arrow highlights a 45,X sample with SRY/β-globin ratio above the positive threshold of 0.1. Because the Y chromosome was not detected using microarray analysis in this sample and SRY FISH could not be performed because of blood sample unavailability, this result may be either a false positive or a true positive where real-time PCR may be a more sensitive approach for detection of low level mosaicism. For comparisons with 46,XY controls ### *P* < 0.001 for SRY cioy munber and 46,XX controls *** *P* < 0.001 for MS-QMA MR, nonparametric Mann–Whitney test for median was used. FISH, fluorescence in situ hybridisation; MR, methylation ratio; MS-QMA, methylation specific quantitative melt analysis; PCR, polymerase chain reaction; SRY, sex determining region Y.

For venous blood DNA, only samples with three or more copies of X chromosome showed MS-QMA MR above the 0.37 threshold (Fig. 2a). As with our previous FREE2 methylation analyses using the EpiTYPER system (Ref. 8), the 47,XXX and 49,XXXXY groups showed MS-QMA MR significantly above those of female controls. As expected, saliva and venous blood DNA samples with a Y chromosome according to previous laboratory testing also showed ~1 copy of SRY, whereas samples with two or more copies of a Y chromosome showed SRY copy number between 1.5 and 2 (Figs 1b and 2b). The only exception was one 45,X sample that consistently showed SRY/β-globin ratio above the positive threshold of 0.1 (Fig. 2b). Because the Y chromosome was not detected using microarray analysis in this sample and SRY fluorescence in situ hybridisation (FISH) could not be performed because of blood sample unavailability, this result may be either a false positive or a true positive where real-time PCR may be a more sensitive approach for detection of low level mosaicism.

### Detection of skewed X-inactivation and the comparison with the EpiTYPER system

**Figure 3.**
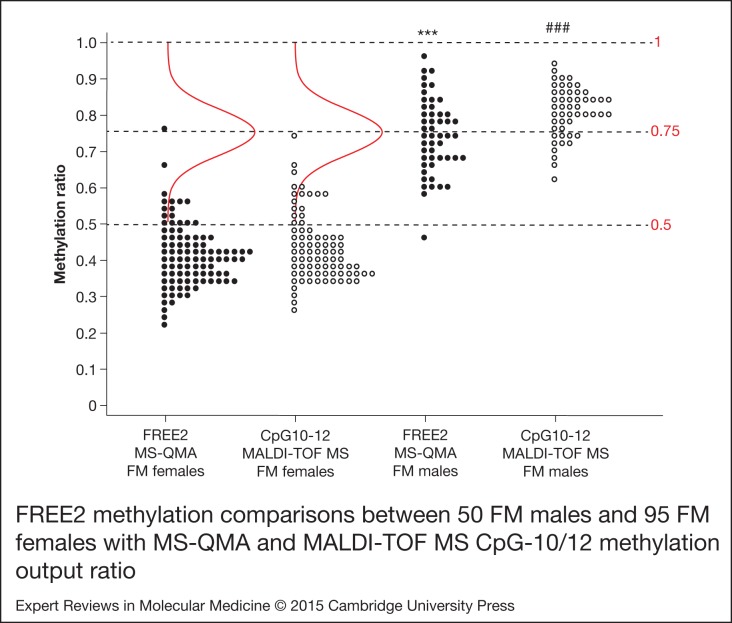
FREE2 methylation comparisons between 50 FM males and 95 FM females assessed with MS-QMA and MALDI-TOF MS MS-QMA targets seven CpG sites within *FMR1* intron 1 including CpG10-12 and three sites within exon 1; whereas MALDI-TOFMS CpG10-12 MOR is specific only for methylation of CpG10-12. Note: all comparisons between FM males and FM females showed *P* < 0.001; ^∗∗∗^ comparisons for MS-QMA mean values, with two-sample t-test used as the data were normally distributed; ^###^ comparison for MALDI-TOF MS between median values of the FM groups, with nonparametric Mann–Whitney test used because of the data not being normally distributed. The bell shaped curve represents the expected normal distribution for the FM females methylation values if the X-inactivation at the locus were random, with mean methylation ratio of 0.75, the higher tail of distribution at 1 and the lower tail of distribution at 0.5. FM, full mutation; *FMR1*, Fragile X mental retardation 1; FREE2, fragile X related epigenetic element 2; MALDI-TOF MS, Matrix-assisted laser desorption/ionisation time of flight mass spectrometry; MS-QMA, methylation specific quantitative melt analysis; MOR, methylation output ratio.

Here we have explored the relationship between X-inactivation and FREE2 MS-QMA MR in a larger sample than was previously analysed with the EpiTYPER system (Ref. 6). Despite the differences in assay design, there was significant correlation between the two assays in blood and saliva of female controls, blood of FM males and females and in saliva of 47,XXY individuals (Table 1). The distribution of the MR values in venous blood DNA for both assays was almost identical in FM females. It showed clear skewing towards the unmethylated state from the distributions expected if the X-inactivation were random at this locus (Fig. 3; bell shaped curve). For both assays, methylation in FM males was significantly elevated compared with that in FM females. The only difference between the two assays was evident in FM males where for the EpiTYPER system the methylation ratio was skewed towards 1, whereas the FREE2 MS-QMA MR was normally distributed in the same samples, with the top and bottom tails of distribution at ~1 and 0.5, respectively.

**Table 1.** Simple linear regression analysis between fragile x related epigenetic element 2 (FREE2) methylation assessed using matrix-assisted laser desorption/ionisation time of flight massspectrometry (MALDI-TOF MS) – the predictor variable, and methylation specific quantitative melt analysis (MS-QMA) – the outcome variable, in venous blood and saliva DNA.

	Venous blood DNA		Saliva DNA			
	*n*	Coef ± SE	*R* ^2^	*n*	Coef ± SE	*R* ^2^
♂ controls	17	0.125 ± 0.340	0.01	13	−0.500 ± 1.158	0.02
♀ controls	86	0.256 ± 0.115*	0.06	26	0.440 ± 0.208*	0.16
♂ FM	49	0.559 ± 0.069***	0.58	…	…	…
♀ FM	95	1.040 ± 0.073***	0.69	…	…	…
47,XXY	…	…	…	47	0.330 ± 0.130*	0.12

FM, full mutation

*n* represents sample size; Coef. represents regression coefficient; SE represents standard errors

Note: Regression results are presented for groups with *n* > 10; *P < 0.05; **P < 0.01; ***P < 0.001.

## Discussion

In this study FREE2 MS-QMA identified all SCAs when combined with an SRY test. It also detected both locus-specific skewing towards the hypomethylated state, as is apparent in FM females, and skewed X-inactivation towards the hypermethylated state for the whole X-chromosome, as with SCAs with three or more X chromosomes. Continuing from the previous observations with venous blood and saliva DNA where the EpiTYPER system FREE2 CpG10-12 methylation analysis detected skewed X-inactivation (Ref. 6) and identified SCAs when combined with SRY analysis (Ref. 8), we assessed the performance of MS-QMA for detection of these same abnormalities. We found that as with the EpiTYPER system, when combined with the SRY analysis at the MR threshold of 0.1, MS-QMA identified SCAs with specificity and sensitivity approaching 100%. However, without the SRY analysis and at the maximum control female methylation threshold of 0.37, MS-QMA could not detect the vast majority of SCAs using either venous blood or saliva DNA. The exceptions were samples with three or more X chromosomes that showed median MR significantly higher than the median of the female control range. Because in these samples only one X chromosome remains active, with more X chromosomes in the ‘DNA mix’, skewing towards the hypermethylated state was observed as be expected, using MS-QMA in this study and the EpiTYPER system previously (Ref. 6).

In the *FMR1* locus specific context, as with the EpiTYPER system results, the MS-QMA MR control range was lower than the expected value of 0.5, with ~0.4 being the maximum control value, and ~0.3 being the mean value. This is an unusual feature of the FREE2 region that we have reported earlier using the EpiTYPER system, but could not explain (Ref. 6). However, we have suggested that in part because of this property we could identify cognitively impaired hypermethylated FM females more readily than the analysis of CpG island region targeted by Southern blot as part of the standard diagnostic protocol (Ref. 4).

In this study, MS-QMA also showed clear skewing towards the unmethylated state from the distributions expected if the X-inactivation were random in FM females at this locus. Specifically, if the X-inactivation were random, one would expect the higher tail of the MR distribution to be at 1, representing all cells having the normal size allele on the inactive X and the methylated FM allele on the active X. The lower tail of the distribution would then be 0.5 MR with all cells having the FM alleles on the inactive X and normal cell alleles on the active X. In contrast, the upper tail for both the MS-QMA and EpiTYPER system results was at ~0.7 MR with the lower tail at ~0. 2MR, demonstrating skewing towards the unmethylated state. Because in FM males more than half of the samples showed MR values between 0.7 and 1 MR using either method, the lack of any FM female results with MR values above 0.7 cannot be considered to be a result of technical bias associated with the MS-QMA assay. The most likely explanation for this is biological, possibly in vivo selection for cells at the lower tail of the MR distribution that express Fragile X Mental Retardation Protein (FMRP) (Ref. 6). This is also consistent with the previous reports of FM females being generally less severely affected than FM males, because of the residual *FMR1* activity (Ref. 21).

It is also interesting to note that for FM males with 100% methylation at the *FMR1* CpG island (shown on Southern blot), both MS-QMA and the Epityper system find methylation of *FMR1* intron 1 between 60 and 100%. This suggests that methylation mosaicism between different CpG sites in FM males may be more common than previously thought, and this may relate to the wide spectrum of clinical phenotypes (other than severe cognitive impairment) found in FM males. It also suggests that the diagnostic classification of methylation mosaicism in FXS needs to be re-defined to include methylation of more than just a few restriction sites targeted by Southern blot.

### Comparisons with other HRM based methods for quantitative methylation analysis

In the context of FXS we are aware of only two methods utilising HRM (Refs 22, 23) with no publications on HRM applications for detection of SCAs and XCI. Furthermore, previous methods utilising HRM for FXS specific methylation analysis of *FMR1* alleles were only applied to venous blood and not saliva DNA (Refs 22, 23). Although these HRM methods worked as expected to identify FM males and in spiked samples using mixtures of methylated and unmethylated DNA, generating melting peak standard curves, they did not provide quantitative information that could be used to effectively differentiate FXS females with FM from normal size control and PM carrier females. This could be either because the HRM assays targeted different biomarker sites to FREE2, and methylation of these other sites was too variable in the female control methylation range, and/or because the technical variation in quantitative methylation was too great in this intermediate methylation range.

To overcome the technical limitation of quantitative HRM for detection of FXS FM females, we found that inter and intra run methylation ratio variation has to be ~5% in the methylation range between 30 and 60%. We have achieved this using a quality control filter process involving quantitative real-time PCR analysis of serial dilutions after bisulphite conversion. We hypothesise that by diluting the converted samples we were also diluting out the impurities which could interfere with the downstream quantitative HRM assessment at any specific melting temperature. This procedure minimised the ‘noise’ that was otherwise associated with the downstream HRM based methylation assessment. Without these serial dilutions and the filter process described here, the HRM AFU values could not be used to differentiate FXS FM females from the controls, or detect skewed X-inactivation in blood and saliva.

In summary, we have demonstrated the more immediate applications of MS-QMA for the detection of skewed XCI at the *FMR1* locus and in different types of SCA. However, the method has potential for any application where quantitative detection of even small changes in the genomic position and the amount of locus specific methylation is of diagnostic or prognostic significance because of mosaicism in the methylation state within and between different cell types (Ref. 3). These may include monogenic disorders such as Rett Syndrome (Ref. 24), trinucleotide disorders such as Friedreich ataxia and myotonic dystrophy (Refs 3, 25, 26, 27), imprinting disorders such as Angelman, Prader-Willi and Beckwith-Wiedemann Syndromes (Ref. 28), disorders related to more general skewed XCI (Refs 29, 30), as well as somatic genetic disorders such as cancer (Refs 1, 2).

## Supplementary material

For supplementary material accompanying this paper visit http://dx.doi.org/10.1017/erm.2015.11. click here to view supplementary material
