# Supplementary material for: Detection of skewed X-chromosome inactivation in Fragile X syndrome and X chromosome aneuploidy using quantitative melt analysis
Source: Expert Rev Mol Med. 2015 Jul 1;17:e13. doi: 10.1017/erm.2015.11 (PMC4836209; doi:10.1017/erm.2015.11)
Supplement: Supplementary file 1 [file S1462399415000113sup001.docx]

**Glossary**

AFU - arbitrary fluorescence units

FISH - fluorescence in situ hybridization

FM - full mutation

FREE2 - Fragile X Related Epigenetic Element 2

FSIQ - full scale intelligence quotient

FXS - Fragile X syndrome

HRM - high resolution melt

MALDI-TOF MS - Matrix-assisted laser desorption/ionization time of flight mass spectrometry

MOR - methylation output ratio

MR - methylation ratio

MS-QMA - Methylation Specific Quantitative Melt Analysis

PCR - polymerase chain reaction

PIQ - performance intelligence quotient

PM - premutation

PM/FM mosaic – mosaic for combination of cells with permutation and full mutation alleles

QC - quality control

SCA - sex chromosome aneuploidies

SRY - sex determining region Y

VCGS - Victorian Clinical Genetics Services

VIQ - verbal intelligence quotient

XCI - X chromosome inactivation
